# A System Out of Breath: How Hypoxia Possibly Contributes to the Pathogenesis of Systemic Sclerosis

**DOI:** 10.1155/2011/824972

**Published:** 2011-11-20

**Authors:** T. W. van Hal, L. van Bon, T. R. D. J. Radstake

**Affiliations:** ^1^Department of Rheumatology, Radboud University Nijmegen Medical Centre, 6500 HB Nijmegen, The Netherlands; ^2^Department of Rheumatology, Nijmegen Center for Molecular Life Sciences (NCMLS) and Nijmegen Institute for Infection, Immunity and Inflammation (N4i), Radboud University Nijmegen Medical Centre, Geert Grooteplein 8, 6500 HB Nijmegen, The Netherlands

## Abstract

Systemic sclerosis (SSc) is an autoimmune disease characterized by vascular alterations and immunological disturbances and fibrosis, the order of which remains to be fully determined. Clinically, patients show clear signs of hypoxia in skin and internal organs. The low oxygen tension is potentially caused by a yet to be indentified circuitry involving the three features that typify SSc. In addition, once present, the hypoxia creates a vicious circle of ongoing pathology. In this paper, we provide an overview of the evidence that points towards the mechanisms causing hypoxia in SSc. In addition, data that suggest how hypoxia itself may orchestrate worsening of symptoms is presented. Altogether, it is clear that hypoxia is an important hallmark in SSc patients. By providing an overview of the mechanisms at play and the possible therapeutic avenues that have emerged, we hope to stimulate researchers to provide novel clues into the conundrum in SSc patients.

## 1. Introduction

**Figure 1:**
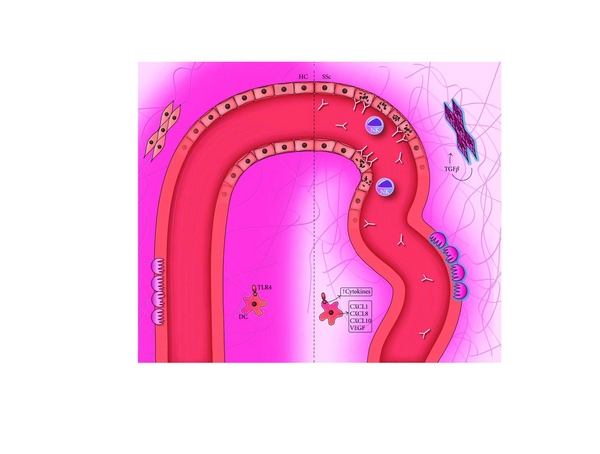
Hypoxia in the pathogenesis of systemic sclerosis. The left side illustrates the normal situation, with a healthy blood vessel delivering oxygen to the surrounding tissue. The right side represents the situation in SSc, where the diseased vessel and overwhelming deposition of collagen fibers prevent the oxygen from reaching the periphery. In the vessel, endothelial apoptosis as a result of antibody-dependent cytotoxicity is visible. Surrounding the ECs, activated pericytes are responsible for the deposition of collagen. The resulting hypoxia leads to a higher cyto- and chemokine production by DCs, in part triggered by TLR stimulation, and to a continuing loop of TGF*β* production, collagen synthesis, and myofibroblast differentiation of fibroblasts. This in turn leads to a hindered dispersion of oxygen, keeping the vicious circle going on.

Systemic sclerosis (SSc) is typified by vascular alterations and immunological disturbances and fibrosis of the skin and internal organs, which culminates in severe disabilities and not seldom premature death. Although the abovementioned pathways are all clearly involved, their sequel and relative contributions are still a matter of debate. About 90% of the patients diagnosed with SSc experienced Raynaud's phenomenon long before the appearance of other clinical symptoms that drives the patient to visit a physician [1]. The diagnosis is often made when patients suffer from a full-blown SSc with rarefaction of the small capillaries as identified by capillaroscopy, digital ulcers, and progressive fibrosis of the skin [2, 3]. Both Raynaud's phenomenon and the rarefaction of capillaries suggest the presence of hypoxia during certain stages of disease. In line with these thoughts, several studies demonstrated a lowered oxygen pressure in SSc skin [4–6]. Data showing a lower pO_2_ only in the lesional SSc skin and a correlation between skin thickness and pO_2_ suggests a direct connection between fibrosis and tissue hypoxia. Lastly, evidence of increased oxidative stress, thought to be caused by ischemia-reperfusion events, is convincing [7, 8]. The precise contribution of the clinically clear hypoxia to the cellular derogatory mechanism is exposed bit by bit. Next to this aberrant regulation of oxygen system, a role for the dysregulated immune system is illustrated by the fact that 75–90% of patients have ANA positivity, mostly explained by the presence of anticentromere or antitopoisomerase antibodies (reviewed in [9]). Recent literature shows the potential interaction between the vascular alterations and the immunopathology found in SSc. The rarefaction of the small vessels clearly lowers oxygen pressure in the skin, and there are signs of increased oxidative stress, which both are able to alarm and activate the plethora of different cell types of the immune system (Figure 1). 

### 1.1. HIF-1*α*

As a result of the hypoxia in skin and other affected tissue in SSc, activation of important hypoxia pathways is plausible. The identification of hypoxia-inducible factor 1 (HIF-1) unlighted the cellular mechanism of oxygen homeostasis. As a transcription factor of genes important in the metabolism of the cell but also angiogenesis, apoptosis, proliferation, and matrix production, this factor seems to play a more then central role in the cellular response to changing oxygen pressures (reviewed in [10]). HIF-1*α* is the tightly regulated form of HIF-1, which is quickly hydroxylated and degraded in normoxic conditions by prolyl hydroxylases [11]. But in case of hypoxia, the levels of HIF-1*α* increase dramatically [12]. Counterintuitive however, HIF-1*α* was found to be decreased in the epidermis of SSc patients compared to healthy controls [6]. As nicely suggested in the review by Beyer et al. [13], this could be caused by a negative feedback loop causing an increase in prolyl hydroxylases resulting in a faster degradation of HIF-1*α*. The persistent upregulation of VEGF (further discussed below) in SSc illustrates the activation of pathways sensitive to oxygen pressure and thus the low expression of HIF-1*α* must be compensated by other factors like HIF-2*α* and HIF-3*α*. The presence of these factors in SSc is currently unknown, and, moreover, the function of these factors is not mutually exclusive with HIF-1*α* [14]. Furthermore, a French cohort SSc patients showed an increased presence of a genetic variant of HIF-1*α*, implying a role in SSc pathogenesis [15]. How a defective HIF-1*α* regulation is precisely involved in the development of SSc will be discussed in detail later in this paper.

## 2. Vasculopathy

Of the three pathogenic features in SSc, vasculopathy is thought to be the first one to occur [16]. The vascular defects can be made visible with nailfold capillaroscopy, showing giant capillaries, loss of architectural arrangement, hyperpermeability, and dropout of capillaries [2]. The lower density and quality of vessels leads to a reduced blood flow and consequential tissue hypoxia. 

 The absence or aberrant function of dermal endothelial cells (ECs) is one mechanism often addressed to explain the onset of vasculopathy. ECs can be distinguished on immunohistochemistry by the expression of CD31, von Willebrand factor (vWF), and vascular endothelial cadherin (VE-cadherin). Dermis of early diffuse SSc patients (disease duration <2 years in 27/30 patients) shows a population of CD31+ endothelial cells with no expression of vWF and VE-cadherin [17]. Another group observed apoptotic vWF positive cells in skin section from early diffuse SSc patients, indicating apoptosis of EC. In the UCD 200/206 chicken SSc model, this apoptosis of ECs was already present before the appearance of perivascular infiltrates or fibrosis [16] and was also identified in the lungs, esophagus, and kidneys [18]. The presence of immunoglobulins (Ig) on these ECs [16] points towards a role of antiendothelial cell antibodies (AECA), which are known to be present in the serum of SSc patients [19]. The induction of EC apoptosis is thought to be the result of antibody-dependent cellular cytotoxicity [20, 21], and Sgonc et al. have suggested that natural killer (NK) cells form a likely candidate to initiate this process [22]. 

Next to the increased loss of ECs due to apoptosis, there might also be a problem in endothelial repair in SSc. SSc patients show a decreased amount of bone-marrow (BM) resident endothelial progenitors, while the hematopoiesis of other cell lines seems to be undisturbed [23]. The decreased amount of BM resident endothelial precursors could be a consequence of the above-mentioned AECA, which can induce apoptosis of the precursors upon exposure to AECA-positive serum [23]. Another mechanism to be considered is a higher efflux of these progenitor cells to the periphery, possibly as a result of elevated VEGF levels (as discussed in the next paragraph) [24]. Of interest in this light are the articles showing an increased amount of circulating endothelial progenitors cells (CEP) [25, 26], although there are also groups reporting a conflicting lower number of CEP [27]. These latter are more in line with a hampered recruitment of the BM progenitor cells to the periphery. There was no difference between patient and healthy control CEP when looking at VE-cadherin, CD31, and vascular endothelial growth factor receptor 2 (VEGFR-2) [27, 27]. Nevertheless, the capacity to differentiate in vitro to EC is diminished in SSc progenitors, as shown by a tempered induction of vWF after prolonged culture [27]. On the other hand, healthy control (HC) and SSc CEP show an equal ability to form tubules when cultured on a Matrigel matrix in vitro [28]. Thus, in principal, SSc CEP should be able to form coherent and functional vessels.

In normal circumstances, hypoxia induces the expression of angiogenic factors to stimulate angiogenesis. One of the key angiogenic growth factors is vascular endothelial growth factor (VEGF), which is produced by, among others, fibroblasts in response to HIF-1*α* [6]. Despite the low presence of HIF-1*α* in SSc, the expression of VEGF is increased in serum and skin of SSc patients [6, 29]. The ongoing induction of VEGF must then be caused by another mechanism, like the effect of the inflammatory cytokines interleukin 1 (IL-1) and platelet-derived growth factor (PDGF). These are highly present in SSc [30, 31] and can indeed induce the expression of VEGF in fibroblasts [6]. 

The effects of VEGF are regulated by its receptors, VEGF receptor 1 and 2 (VEGFR-1 and VEGFR-2). From these, VEGFR-2 is the effecting molecule and VEGFR-1s main function is to regulate the phosphorylation and activation of the other [32]. In scleroderma skin, both receptors are found to be higher present with an intenser staining for VEGFR-2 than for VEGFR-1 [6]. Exposure of EC to hypoxia *in vitro* showed a downregulation of VEGFR-2 and upregulation of VEGFR-1 in HC and SSc samples; the latter showed a lower expression in SSc EC [28]. This differential regulation can make the cells more responsive for VEGF. Correct timing of VEGF upregulation results in recruitment of EPC and formation of new vessels, which improves the oxygen supply to the tissue. In contrast, an ongoing VEGF stimulus caused by a higher VEGF and a defective regulation due to a relative decrease in VEGFR-1 elicits the formation of aberrant vessels [33], resembling the anomalous vessels seen on nailfold capillaroscopy in SSc [2].

In conclusion, in SSc, the vascular reaction to hypoxia is dominated by the growth factor VEGF. On the other hand, there is the altered quantity and phenotype of the ECs and their progenitors, which could be a causal factor in the appearance of hypoxia in SSc. The fact that this altered phenotype is not present in the EC of patients who were treated with intensive immunotherapy and subsequent stem cell transplantation is a promising thought [17]. This could imply reversibility of the vascular defects and might open novel avenues for therapeutic intervention.

## 3. The Dysfunctional Immune System

It is by now generally accepted that hypoxia can induce inflammation and that inflammation itself causes hypoxia in tissues. The decrease in oxygen tension during inflammation is a logic result of the increasing metabolic demand of cells, thrombosis, and compression of the vessels due to edema (interstitial hypertension). The effect of hypoxia, causing a proinflammatory environment throughout the body, is nicely illustrated by individuals that spend time at extreme height and display increased levels of IL-6 and CRP [34]. Furthermore, we know from transplantation medicine that the expression of Toll-like receptors (TLRs) and pro-inflammatory cytokines correlates with the amount of ischemia [35, 35, 36]. 

The main TLR-expressing cells are the cells of the innate immune system, especially the antigen-presenting cells. In response to the hypoxic environment during inflammation, these cells need to increase their expression of HIF-1*α* for a proper function. This was elegantly demonstrated in phagocytes from HIF knockout mice that could not efficiently remove bacteria and get persistent ulcers [37, 38]. Differentiation and maturation of dendritic cells (DCs) is inhibited under hypoxic conditions [39] but they showed an increased production of CXCL1, VEGF, CXCL8, and CXCL10 [40, 41], all of which increased in SSc [29, 42, 43]. In contrast with these latter observations, these hypoxic DCs showed a lowered CCL2 and CCL18 production [40, 41], two pivotal chemokines that are repeatedly found to be increased in the circulation of SSc patients [43, 44]. Interestingly, other research showed an increased DC maturation and costimulatory capacity after TLR ligation during hypoxia [45, 46], suggesting that these cells are already in an activated state. Taking into account the endogenous TLR4 ligands found in SSc serum [47], the increased cytokine production in response to TLR ligands [48], and the increase in circulating cytokines mainly produced by these antigen presenting cells, it is tempting to speculate about a role for the hypoxic environment in this condition and this justifies further research to unravel this conundrum.

The effects of lowered oxygen pressure on the adaptive immune system are also quite evident. Hypoxia increases the expression of IL-10, TGF-b, galectin-1, and Foxp3, all supporting the development of regulatory T cells (Tregs) in hypoxic conditions. This is in line with several studies that reported increased numbers of Tregs in the circulation of SSc patients [49–51]. HIF-1*α* expression in T lymphocytes enhances the apoptotic rate and decreases T-cell function. In line with this, conditional deletion of HIF-1*α* in T and B cells is associated with the appearance of autoimmune responses in mice [52]. More intriguingly, the HIF-1*α* deficient T lymphocytes produced more pro-inflammatory cytokines in response to TCR triggering than HIF-1*α* expressing control cells [53, 54]. Taken into account the low HIF-1*α* expression in SSc patients, even in low oxygen conditions, it is tempting to suggest that the function of T lymphocytes in SSc is diverted by their impaired response to the hypoxic condition. Altogether, a hypoxic environment as present in SSc patients has clear effects on the immune system which might be augmented by an inherent defect to adept to low oxygen concentrations by SSc cells. Research focused on the effect of hypoxia on immune cells from SSc patients however is scarce and needs more attention in future investigations. Next to that, the underlying circuitry that explains this possible altered response to hypoxia by SSc cells remains elusive and warrants further investigation. 

## 4. Fibrosis

Fibrosis is regarded to be the end stage of SSc and is often thought to cause the majority of the clinical symptoms. It is no wonder that most of the research of the last decades in this field focused on fibroblast biology and hypoxia as a causative factor for the extensive extracellular matrix (ECM) deposition.

A lower supply of oxygen to the tissue can be caused by vessel depletion or impaired diffusion. Where vasculopathy can be the cause of the former, fibrosis can lead to the latter. An important effector cell in this perivascular fibrosis is the pericyte. Pericytes can be found together with ECs around capillaries and show characteristics resembling vascular smooth muscle cells (SMCs) [55, 56]. In the perivascular area in SSc skin, pericytes with a myofibroblast phenotype have been observed, expressing alpha smooth muscle actin (*α*-SMA) and ED-A splice variant fibronectin (ED-A FN). These cells also show expression of the cell-surface glycoprotein Thy1 [57], which is essential in the differentiation of fibroblasts to myofibroblasts [58]. The upregulation of *α*-SMA in pericytes is associated with collagen production [59]. Furthermore, a reduced endothelial growth was observed when combining pericytes and EC in culture [56]. Taking together, these myofibroblast-like cells can lead to both perivascular fibrosis and endothelial dysfunction and thus cause a reduced supply of oxygen to the tissue.

The excessive production of ECM components by fibroblasts in reaction to transforming growth factor beta (TGF*β*) and connective tissue growth factor (CTGF) is a paradigm in scleroderma [60]. Lately, there seems to be a paradigm shift noting that this excessive ECM production is not only due to an intrinsic defect in the fibroblasts, but also in part caused by a normal reaction of these fibroblasts to the pathological environment present in SSc patients. Indeed, in hypoxic circumstances, fibroblasts from SSc patients and HC react in the same profibrotic way. Exposure of fibroblasts to a low oxygen environment leads to HIF-1*α*-dependent upregulation of both TGF*β* and CTGF [61–63]. Stabilization of HIF-1*α*, as occurs during hypoxia, also elevates the sensitivity of cells towards TGF*β*, leading to a quicker TGF*β*-dependent upregulation of CTGF. Herein could lay the explanation for the heightened serum CTGF in SSc [62]. Moreover, during hypoxia, the fibroblasts show an upregulation of genes involved in ECM synthesis and regulation, including fibronectin, thrombospondin, pro*α*2(I) collagen (COL1A), and lysyl hydroxylase 2 (LH2) [64, 65]. This upregulation is in part regulated by HIF-1*α*, but totally dependent on TGF*β* [64]. Last but not least, the hypoxic damage and oxidative stress could induce tissue damage and the subsequent release of associated molecular patterns (DAMPs), which could in turn stimulate TLRs on the fibroblasts and thus lead to activation [66].

Epithelial-to-mesenchymal transition (EMT) is a novel concept in fibrosis of lung and kidney, two severe complications of SSc. It describes a process in which epithelial cells develop mesenchymal characteristics, such as an increased expression of *α*-SMA and vimentin and a decrease in expression of E-cadherin. TGF*β* is the prototypic stimulus of EMT [67]. EMT can also be induced by a lower oxygen pressure, as is shown by hypoxic culture of alveolar and kidney tubulus epithelial cells. The role of HIF-1*α* and TGF*β* in this transition seems to be dependent on the origin of the epithelial cells. In alveolar cells, both molecules are required for EMT, while in kidney epithelium the process seems to be independent of these two proteins [4, 61]. Recently, EMT has been observed in the skin of a murine SSc model, suggesting that this process may be involved in both dermal and pulmonal fibrosis [68].

In contrast to the long-thought intrinsic defect in fibroblasts, data show that these cells respond in a normal way to abnormal circumstances. Taking the above mentioned together, there is evidence that in SSc hypoxia can lead to differentiation and activation of fibroblasts but might have similar effects on primary immune cells all contributing to an extensive ECM deposition. 

## 5. Conclusion 

Most human cells are able to adapt to a broad range of oxygen tension varying between 40–100 mmHg in the circulation and 4–20 mmHg in tissue. In contrast to these physiological differences, in certain condition pathological hypoxia develops. In SSc patients pathological hypoxia is obvious only by looking at their hands and seeing the digital ulcers patients suffer from. The response to this loss of oxygen has to be well coordinated and tightly regulated by factors like HIF-1*α*. The hypoxia caused by a combination of activated immune cells (increasing metabolic demands), rarefaction of blood vessels, and perivascular fibrosis becomes detrimental without this regulation. In SSc, there is no sign of increasing HIF-1*α* activity and therefore the low oxygen tension can go on activating fibroblasts (etc.), aggravating immune responses, and impairing endothelial cells and thereby further increasing the hypoxic environment. Moreover, the ongoing hypoxia will increase TGF*β* levels and will therefore increase the production of extracellular matrix, further increasing fibrosis and thus the distance to the closest blood vessel [64, 69, 69]. Interestingly, another detrimental effect of hypoxia is shown in cancer research by the profibrotic and proinflammatory effect of the increased reactive oxygen species and autophagy caused by low oxygen tension [70]. Eventually, it seems inescapable that the system will choke in its own vicious circle. As shown by the effect of stem cell therapy [17], there are options to interfere in this ongoing process. For example, one would be able to interfere in the impaired response to hypoxia more specifically by the use of propyl-hydroxylase inhibitors (reviewed in [71]). In addition, the pharmacological increase of bilirubin by the use of atazanavir could directly provide more anti-inflammatory agents thereby bypassing the dysfunctional hypoxic pathway [72]. These therapeutic interventions could be given systemically but, alternatively, could also be specifically targeted to certain cells of the immune cells by exploiting recent knowledge on liposomes or even nanoparticles. In conclusion, the research field on therapeutic interventions in detrimental hypoxia is growing which will hopefully lead to the broadening of our therapeutic armamentarium to treat this disease.
